# Using the information embedded in the testing sample to break the limits caused by the small sample size in microarray-based classification

**DOI:** 10.1186/1471-2105-9-280

**Published:** 2008-06-14

**Authors:** Manli Zhu, Aleix M Martinez

**Affiliations:** 1 Department of Electrical and Computer Engineering, The Ohio State University, Columbus, OH 43210, USA; 2 Department of Biomedical Engineering, The Ohio State University, Columbus, OH 43210, USA

## Abstract

**Background:**

Microarray-based tumor classification is characterized by a very large number of features (genes) and small number of samples. In such cases, statistical techniques cannot determine which genes are correlated to each tumor type. A popular solution is the use of a subset of pre-specified genes. However, molecular variations are generally correlated to a large number of genes. A gene that is not correlated to some disease may, by combination with other genes, express itself.

**Results:**

In this paper, we propose a new classiification strategy that can reduce the effect of over-fitting without the need to pre-select a small subset of genes. Our solution works by taking advantage of the information embedded in the testing samples. We note that a well-defined classification algorithm works best when the data is properly labeled. Hence, our classification algorithm will discriminate all samples best when the testing sample is assumed to belong to the correct class. We compare our solution with several well-known alternatives for tumor classification on a variety of publicly available data-sets. Our approach consistently leads to better classification results.

**Conclusion:**

Studies indicate that thousands of samples may be required to extract useful statistical information from microarray data. Herein, it is shown that this problem can be circumvented by using the information embedded in the testing samples.

## Background

The emergence of modern experimental technologies, such as DNA microarray, facilitates research in cancer classification. DNA microarrays offer scientist the ability to monitor the expression patterns of thousands of genes simultaneously, allowing them to study how these function and how they act under different conditions. This can lead to a more complete understanding of molecular variations, in addition to morphologic variations among tumors. A large number of studies have used microarrays to analyze the gene expression for breast cancer, leukemia, colon tissue, and others, demonstrating the potential power of microarray in tumor classification [1-7].

An important open problem in the analysis of gene expression data is the design of statistical tools that can cope with a large number of gene expression values per experiment (usually thousands or tens of thousands) and a relatively small number of samples (a few dozen). This imbalance between number of genes and samples, generally results in *over-fitting *[8], i.e., the problem where one can easily find a decision boundary which separates the training samples perfectly while performing poorly on independent testing feature vectors [9]. This problem has been cited as a major deterrent for the successful use of microarrays technology in prognosis and diagnosis in cancer research [8,10,11].

**Figure 1:**
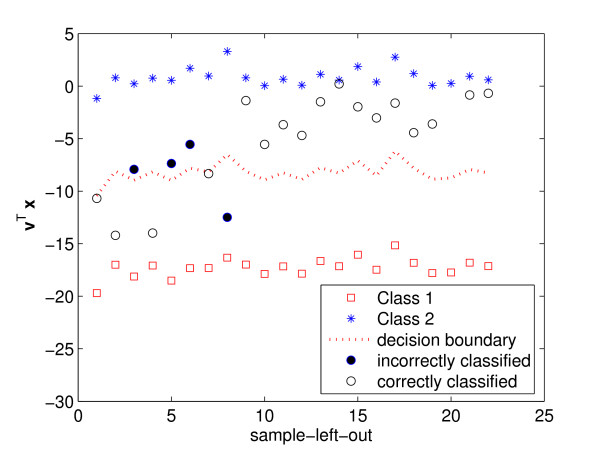
**Plotted here is an illustration of the over-fitting problem. **The *x*-axis indicates the index of the sample left out for testing. In all cases, the training samples are perfectly separated (i.e., all the samples from class 1 and class 2 are projected onto two clearly distinct points – shown as * and □, respectively). The test samples fall in the middle and are not always correctly classified.

In Fig. 1, we show one such example with an application to breast cancer classification. In this example, we have 22 samples. The first 7 are from tumor tissue and the remaining 15 are from normal tissue. To test a typical classification algorithm, it is common to use the leave-one-out cross-validation test [12]. That means, 21 of the samples are used to train the classifier while the remaining sample is used for testing. There are 22 possible ways of leaving one of the samples out for testing, each producing a possible outcome. In Fig. 1, the *x*-axis represents the index of the sample left out (1 to 22). The *y*-axis shows the resulting projection onto the one-dimensional space found by Fisher's Linear Discriminant Analysis [13] (LDA), which is known to be among the best algorithms in such classification problems [11,14]. The sample vectors of the tumor class are projected onto this one-dimensional space and marked with the star symbol (*). The projection of the samples belonging to the non-tumor category are shown as squares (□). We note that LDA *perfectly classifies all of the 21 training samples*, since all the cancer sample vectors are projected onto exactly the same position while the non-tumor samples are projected onto a single separate location. Next, to classify new, independent testing feature vectors, it is common to use the nearest mean approach, where the testing sample is projected onto our one-dimensional space and classified according to the label of the nearest class mean. This classifier is shown by the dotted line in Fig. 1. The feature vectors previously left out for testing are shown in the figure as circles. Filled circles indicate misclassifications. Open circles correct classifications. Several of the testing samples are incorrectly classified, because the discriminant information encoded in the training samples is not the same as that found in the testing one, i.e., the classifier is over-fitted to the training set.

### Over-fitting

Over-fitting can be solved by collecting more samples, but recent results predict hundreds, if not thousands, of samples would be necessary to resolve this issue [15,16]. Unfortunately, in many studies, such a large number of samples is prohibitive, be it due to cost (time, economical) or limited access to patients in rarely occurring cancers.

The most common strategy to overcome these difficulties and avoid over-fitting is to reduce the dimensionality of the original space by choosing a subset of genes that can (theoretically) discriminate tumor tissue from normal; i.e., pre-selection of genes. These pre-selected genes may have explicitly biological meaning or implications in the molecular mechanism of the tumorigenesis [17,18]. Their objective is to increase the classification accuracy, decrease the computation cost of the classifier and clarify the biological interpretation of cancers. A variety of gene selection algorithms have been proposed for this purpose [1,14,17,18].

Unfortunately, a method for pre-selecting genes that works well on one data-set, will not generally work as expected on another [19]. Further, the results are many times unstable due to the limited amount of data used in pre-determining such a pool of genes [11]. Hence, the results can be biased toward the characteristics of our available data or, even, toward the way this data was collected [20]. This is one of the reasons why biomarkers (genetic markers) and other selection mechanisms do not always generalize to novel experiments [8]. To determine the (complex, underlying) biological process involved in the likelihood of developing a certain cancer, it is necessary to study the relation of each individual gene as well as their combinations, because when combined with others a gene can express itself.

Several methods, such as maximum likelihood [21] (ML), weighted voting [1] (WV), *k*-nearest neighbor [21] (*k*NN), Fisher's Linear Discriminant Analysis [13] (LDA) and Support Vector Machines [22,23] (SVM) are, in principle, capable of dealing with a large number of genes (features), and many are known to generalize to new samples when the training set is very large [24]. However, when the number of features is very large and the number of samples small, these methods cannot avoid the over-fitting problem [9]. It remains a key open problem to define classification strategies that can be applied to a large number of genes while aiming to relieve the influence of over-fitting.

### Current methods

Discriminant algorithms for tumor classification using microarray data were cited above. These correspond to the following.

#### *k *Nearest Neighbor (*k*NN) [21]

In many instances, it is reasonable to assume that observations which are close to each other in the feature space (under some appropriate metric) belong to the same class. The nearest neighbor (NN) rule is the simplest non-parametric decision procedure to adopt this form.

Specifically, the label of a sample **x **is *c *if *C*($\tilde{x}$) = *c*, where

$$\tilde{x}=\arg\underset{x_{i}}{\min}d(x_{i},x),i=1,\cdots ,n,$$

*C*(**x**) denotes the class label of the feature vector **x**, and *d*(·,·) is a distance measurement. Generally, the Euclidean distance is used and (hence) was the one considered in this paper. Notice that this NN-rule only uses the nearest neighbor for classification, while ignoring the remaining pre-labeled data points. If the number of pre-classified points is large, it makes sense to use the majority vote of the nearest *k *neighbors. This method is referred to as the *k*NN rule, and is attractive because it is known to generalize well [24].

#### Weighted Voting (WV) [1]

Applicable for binary classification, where each gene casts a weighted vote for one of the classes, and the final decision is made based on the summation of all individual votes. Specifically, for each gene *g*, a correlation value *P*(*g*) is used for measuring the relation between its expression level and the class distinction. This is defined as *P*(*g*) = [*μ*_1_(*g*) - *μ*_2_(*g*)]/[*σ*_1_(*g*) + *σ*_2_(*g*)], where {*μ*_1_(*g*), *σ*_1_(*g*)} and {*μ*_2_(*g*), *σ*_2_(*g*)} denote the mean and standard deviation of the log of the expression level of gene *g *for each of the two classes. Large values of |*P*| indicate high correlations between gene expressions and class distinction. The vote of each gene is *v*(*g*) = *P*(*g*)(*x*(*g*) - *b*(*g*)), where *b*(*g*) = (*μ*_1_(*g*) + *μ*_2_(*g*))/2, with a positive value indicating vote for class 1 and negative value indicating vote for class 2. The final decision is thus given by

$$C(x)=\{\begin{array}{cc} 1, & \text{if }\sum_{g=1}^{p}v(g)>0,\\ 2, & \text{if }\sum_{g=1}^{p}v(g)\leq 0. \end{array}$$

#### Fisher's Linear Discriminant Analysis (LDA) [13]

LDA is used to find a linear combination of genes where the between-class variance is maximized and the within-class variance (or, equivalently, the covariance matrix) is minimized. Since in microarray data the between-class scatter matrix **S**_*B *_and the covariance matrix Σ_*X *_are both singular, we need to calculate the projection directions **v **from [25]

$$\left[\sum_{i=1}^{p_{B}}\sum_{j=1}^{p_{X}}\frac{\lambda_{B_{i}}}{\lambda_{X_{j}}}(u_{j}^{T}w_{i})u_{j}w_{i}^{T}\right]V=V\Lambda ,$$

where Λ_*B *_= ${\left\{\lambda_{B_{i}}\right\}}_{i=1}^{p_{B}}$ and Λ_*X *_= ${\left\{\lambda_{X_{j}}\right\}}_{j=1}^{p_{X}}$ are the eigenvalues of **S**_*B*_**W **= **W**Λ_*B *_and Σ_*X*_**U **= **U**Λ_*X*_, **W **= ${\left\{w_{i}\right\}}_{i=1}^{p_{B}}$ and **U **= ${\left\{u_{j}\right\}}_{j=1}^{p_{X}}$ are the corresponding eigenvectors, and *p*_*B *_and *p*_*X *_denote the rank of the two matrices. After the projection matrix **V **is obtained, a nearest class mean classifier is used for classification in the sub-space formed by the first *q *columns of **V**. Since the sample-to-dimension ratio is small, it is customary to use only the first row of **V**, that is, the most discriminant direction of LDA, **v**, even if *C *> 2. Then, classification reduces to

$$C(x)=\arg\underset{k}{\min}{\left[{\left(x-\mu_{k}\right)}^{T}v\right]}^{2}.$$

#### Support Vector Machines (SVM) [23]

If the data-set is linearly separable, a linear SVM is a maximum margin classifier. This means, SVM will find that hyperplane that divides the data of one class into one region (say, the positive side of the hyperplane), and the other class in another region (the negative side). While doing this, SVM will guarantee that the distance between the samples of class 1 and class 2 that are closets to this hyperplane is maximized. Considering the following linear classifier

$$f(x)=\langle a,x\rangle +b=\{\begin{array}{ll} \geq 1, & \text{for }x\in \text{class }1\\ \leq -1, & \text{for }x\in \text{class }2, \end{array}$$

it can be shown that maximizing the margin is equivalent to solving the following optimization problem

$$\{\begin{array}{ll} \text{minimize} & J(\alpha )=\sum_{i=1}^{n}\alpha_{i}-\frac{1}{2}\sum_{i,j=1}^{n}y_{i}y_{j}\alpha_{i}\alpha_{j}\langle x_{i},x_{j}\rangle \\ \text{subject to} & \sum_{i=1}^{n}y_{i}\alpha_{i}=0,\alpha_{i}\geq 0,i=1,\cdots ,n. \end{array}$$

The weight vector $a=\sum_{i=1}^{n}\alpha_{i}y_{i}x_{i}$ is a linear combination of the training patterns.

#### Maximum Likelihood (ML)

This is a parametric method. It assumes the distribution form *p*_*k*_(**x**) for each class is a prior known – Gaussian distributions being the most common assumption. The parameters of the distribution are estimated using the training samples. ML assigns the sample **x **to the class which gives the largest likelihood to **x**, i.e., *C*(**x**) = arg max_*k *_*p*_*k*_(**x**). When the samples are Gaussian distributed, **x **~ N(*μ*_*k*_, Σ_*k*_), this results in

$$C(x)=\arg\underset{k}{\min}\{{\left(x-\mu_{k}\right)}^{T}\Sigma_{k}^{-1}(x-\mu_{k})+log|\Sigma_{k}|\}.$$

## Results

We first derive the details of the proposed approach and present each of the algorithm items. Extensive experimental validation is then presented in the testing section.

### Algorithm

The key idea used in this paper, is to take advantage of the discriminant information embedded in the testing sample. Rather than looking for its closest match amongst all the training samples, we can *use *the information of the testing sample to improve the classification process, e.g., to find a better discriminant space in LDA.

The reason why classifiers built on training data generally work poorly on testing data is because the distribution of the training samples does not generally represent that of the testing [9]. In such cases, independent testing samples are treated as passive objects; i.e., it is assumed that the (discriminant) information encoded in the testing sample *cannot *be used because its class is *unknown*. Here, we show that it is actually *possible *to take advantage of the *information embedded in the testing sample*, changing the role of the testing sample from passive to active. We will accomplish this by assigning the test sample to each of the possible classes and then determining which of these "assignments" is the correct one. As mentioned above, this is possible because a discriminant approach will generally work best when the test sample is assumed to belong to the *correct *class. Earlier, we used intuitive argumentation to show this. We will now prove this result formally within the LDA framework, which will be used through out this paper as an illustrative solution (although our solution can be extended to work with other classifiers).

#### Discriminant power

Our solution originates from the discriminant power (DP) of linear discriminant analysis, given by [25]

(1) $$DP=\sum_{i=1}^{p_{B}}\sum_{j=1}^{p_{X}}\frac{\lambda_{B_{i}}}{\lambda_{X_{j}}}{\left(u_{j}^{T}w_{i}\right)}^{2},$$

where ${\left\{\lambda_{B_{i}}\right\}}_{i=1}^{p_{B}}$ and ${\left\{\lambda_{X_{j}}\right\}}_{j=1}^{p_{X}}$ are the eigenvalues of the between-class scatter matrix **S**_*B *_and sample covariance matrix Σ_*X*_, respectively, ${\left\{w_{i}\right\}}_{i=1}^{p_{B}}$ and ${\left\{u_{j}\right\}}_{j=1}^{p_{X}}$ are the corresponding eigenvectors, and *p*_*B *_and *p*_*X *_denote the ranks of these two matrices. The between-class scatter matrix is a metric measuring the separability of samples corresponding to different classes, while the covariance matrix defines the sparseness of the data. The goal is to maximize the first metric while minimizing the second. Here, the sample covariance matrix is defined as

$$\Sigma_{X}=\frac{1}{n}\sum_{i=1}^{n}(x_{i}-\mu ){\left(x_{i}-\mu \right)}^{T},$$

where **x**_*i *_∈ ℝ^*p *^is the *i*^*th *^sample vectors, *p *the number of features (genes), and *μ *the sample mean over all **x**_*i*_. The class covariance matrix is similarly given by $\Sigma_{i}=n_{i}^{-1}\sum_{j=1}^{n_{i}}(x_{i,j}-\mu_{i}){\left(x_{i,j}-\mu_{i}\right)}^{T}$, with **x**_*i*,*j *_the *j*^*th *^sample in class *i*, *μ*_*i *_the sample class mean, *C *the number of classes, and *n*_*i *_the number of samples in the *i*^*th *^class, $n=\sum_{i=1}^{C}n_{i}$. The between-class scatter matrix is then

$$S_{B}=\sum_{k=1}^{C}(\mu_{k}-\mu ){\left(\mu_{k}-\mu \right)}^{T}.$$

*DP *measures how well the classes are separated in the subspace spanned by LDA's solution, **v**. Therefore, the larger the value of *DP *is, the better.

To better understand the role of the *DP *score, let us look back at feature extraction. A classical approach used by researchers to perform dimensionality reduction is the well-known Principal Components Analysis (PCA) algorithm. PCA is concerned with the selection of that linear combination of features (from the original feature representation) which carries most of the data (co)variance. This is readily accomplished by finding the eigenvectors of the covariance matrix Σ_*X*_, i.e., Σ_*X*_**V **= Λ**V**, where the columns in **V **are the eigenvectors and Λ is the diagonal matrix of corresponding eigenvalues. Σ_*X *_is, in effect, the *metric *we have decided to maximize.

Linear Discriminant Analysis (LDA) is in fact an extension of PCA. In LDA, one has two metrics, **A **and **B**. The first metric calculates within-class variances, the second is concerned with between-class variations. Thus, in LDA, the goal is to minimize the metric given by **A **while maximizing that given by **B**, e.g., **A **= Σ_*X *_and **B **= **S**_*B*_. This is then equivalent to the following eigenvalue problem **A**^-1^**BV **= Λ**V**.

Unfortunately, this method does not work well when the two metrics disagree [25], that is, when the solution favored by the first metric **A**, does not agree with that of the second metric **B**. In this case, we say that the two metrics are in conflict. Under such circumstances, knowing which of the two metrics is right turns into a guessing game. Taking an average would even be worse, because generally one of the two metrics is correct [26].

Hence, our next goal is to determine which of the classes, where our test sample can be assigned, will provide the smallest conflict, that is, the largest discriminant score *DP*. That we will show how to efficiently do next.

#### Class fitting

In our framework, we first assign the test feature vector **x **to class *i *and then use LDA to obtain the discriminant subspaces **v**_*i*_, *i *= 1,...,*C*. The discriminant power indices *DP*_*i *_can be computed using (1).

This will indicate how well the data is separated when the test feature vector **x **is assumed to belong to class *i*. When **x **is assumed to belong to an incorrect class, LDA will find it difficult to discern that from the other samples, and *DP*_*i *_will be small. When the test sample is however assigned to the correct class, LDA will find it easier to discriminate between classes and the discriminant value (1) will increase. This means that our approach should reduce to assigning the test sample **x **to that class providing the maximum discriminant power when **x **is assigned to it. Unfortunately, this is not possible, because when the number of genes (features) *p *is much larger than the number of samples *n*, the value of *DP*_*i *_is always 1 regardless of the value of the parameter *i*. This is formally stated in the following.

**Theorem 1. ***Let the number of features (e.g., genes) be $p\geq \frac{n-C}{C-1}$, where n is the number of samples, and C the number of classes. Then, the discriminant power DP for LDA's solution is always equal to one, DP *= 1.

*Proof*. Let Σ_*i *_denote the sample covariance matrix of class *i*, *i *= 1,...,*C*, *p *the dimensionality of the sample feature vectors, *n*_*i *_the number of samples in class *i*, and *n *the total number of samples, $n=\sum_{i=1}^{C}n_{i}$. Since *p *>*n *≥ *n*_*i*_, we have *p *>*n*_*i*_. Hence, *dim*(*null*(Σ_*i*_)) = *p *- *n*_*i *_+ 1. LDA's solution is the intersection of the null spaces of Σ_*i*_. This intersection is non-empty if $\sum_{i=1}^{C}\left(p-n_{i}+1\right)>p$. That is, when □

$$p>\frac{n-C}{C-1}.$$

**Figure 2:**
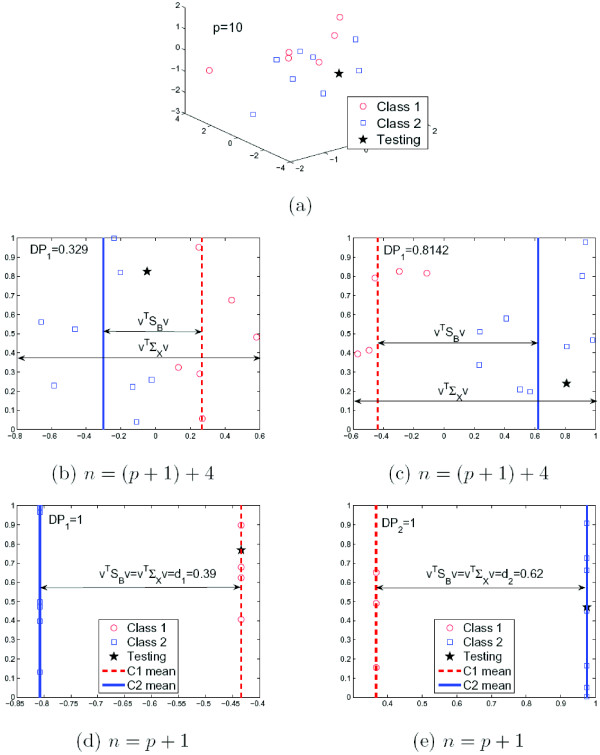
**(a) Shown here are the three dimensions with largest variance of the the randomly generated data in ℝ^10^.** The horizontal axis in (b)-(e) corresponds to the direction **v **found by Fisher's LDA and when assuming the sample vector (star) corresponds to the first class in (b) and (d) and to the second class in (c) and (e). For visualization purposes, the samples have been randomly distributed about the vertical axis in (b)-(e). This helps illustrate the separability of the two classes shown as red (dashed) and blue (solid) lines. We now note that when *n *>*p *+ 1, the value of *DP *is a good measure of separability. When *n *≤ *p *+ 1, *DP *collapses (Theorem 1), and *d*_*i *_becomes the appropriate measure of discriminant ability.

This result is illustrated in Fig. 2 for the case of *C *= 2. In this figure, we synthetically generated *n *samples in ℝ^10 ^for a total of two classes. For visualization purpose, we only show the data using the three dimensions with largest variance in Fig. 2(a). Red circles represent samples from class 1, blue squares for class 2. The black star is the testing sample **x**, which is randomly drawn from class 2 (squares). Next, we project the data onto the direction found by Fisher's LDA when different amounts of training samples *n *are used, Fig. 2(b)–(e). In Fig. 2(b)–(c), we used *n *= 15 = *p *+ 5. That is, we keep the dimensionality of the space *p *(which is 10) smaller than the number of sample *n*, i.e., *p *<*n*. Since there are more samples than features (genes), the discriminant power approach of (1) is applicable. In Fig. 2(b), we calculate *DP*_1_, assuming the test sample belongs to class 1. In Fig. 2(c), we test the alternate hypothesis of **x **actually belonging to class 2, and calculate *DP*_2_. It is clear from the figure that the second option provides a much larger discriminant power and, therefore, the algorithm classifies the test sample in the correct class. However, when the number of samples *n *is smaller than or equal to *p *+ 1, the value of *DP*_*i *_is always one. This is illustrated in Fig. 2(d)–(e), which show the projections when *n *= 11 = *p *+ 1. All samples from class 1 are projected onto a single point and the samples from class 2 to another, *DP*_1_(**x**) = *DP*_2_(**x**) = 1.

#### Final classification

The result above had a purpose beyond that of showing that the discriminant power defined in (1) is inappropriate when *n *≤ *p *+ 1. It is illustrative of the reasons why. First, note that all *DP*_*i *_are equal to one when *n *≤ *p *+ 1, because, in such cases, the projection of each individual class covariance matrix onto the one-dimensional solution found by LDA is always zero. In fact, this is possible because there is always a one-dimensional subspace where all the samples of the same class collapsed onto a single point. This subspaces is the intersection of the null spaces of every class covariance matrix, and was illustrated in Figs. 1 and 2(d)–(e).

Nonetheless, since the projected class covariance matrices are zero, *the between-class variance itself provides the appropriate measure of separability*. We thus denote the distance between classes as that defined by the projected between-class scatter, **v**^*T*^**S**_*B*_**v**.

The framework outlined above, can be readily implemented as follows. First, compute the one-dimensional solution provided by Fisher's LDA when the test sample is assumed to belong to class *k*, **v**_*k*_. That is, **v**_*k *_is obtained using all the training samples and including the test feature vector **x **as an additional "training" sample of class *k*. This solution allows us to compute the discriminant power as

(2) $$d_{k}(x)=v_{k}^{T}S_{B_{k}}v_{k},$$

where $S_{B_{k}}$ is the between-class scatter matrix obtained with all the training samples plus the testing sample **x**.

**Figure 3:**
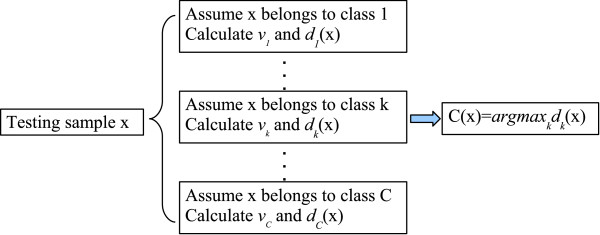
Flow chart of the DP algorithm for the classification of test samples in a *C*-class problem.

The larger the discriminant power (i.e., distance between classes), the better the algorithm can classify the test sample. Hence, the test sample should belong to that class which maximizes (2), that is,

$$C(x)=\arg\underset{k}{\max}d_{k}(x),$$

where *C*(**x**) specifies the class label of the test feature vector **x**. We denote this discriminant, power-based method as DP algorithm. The schematics of the algorithm are illustrated in Fig. 3.

The lower-performance problem in between-class classification [7] is herein solved by taking advantage of the information embedded in the testing feature vector.

### Testing

We have used a variety of databases to validate the algorithm and our claims. This will also serve to prove the superior performance of the proposed approach when compared to the state of the art.

#### Description of the data-sets

##### Breast cancer (BRCA1 and 2)

[4] present a database of human breast cancer with samples generated from 22 primary human breast tumors (7 BRCA1-mutation-positive, 8 BRCA2-mutation-positive and 7 samples from patients with none of the two gene mutations). The interest of the experiment is in determining whether hereditary breast cancers could be classified based solely on their gene-expression profile. The 22 samples are grouped in two ways. The first grouping labels the 22 tumor samples according to *BRCA*1 mutation status (positive or negative), and the second grouping labels the samples according to *BRCA*2 mutation status (positive or negative). There is a total of 3226 genes in this data-set.

##### PROS

This data set is developed to investigate whether gene expression difference is helpful to distinguish prostate cancers with common clinical and pathological features [27]. A total of 102 samples (50 normal and 52 prostate tumor) are included and each sample consists of expression values for 12600 genes. We have normalized the expression levels to a maximum value of 16, 000 and a minimum of 10 to eliminate outliers. The variation filter is then used to exclude genes showing small variation across samples. A 5-fold change variation (Max/Min) and absolute variation of 50 (Max-Min) is applied.

##### PROS-OUT

This data-set is to analyze whether the gene expression data alone can accurately predict patient outcome after prostatectomy [27]. Samples from 21 patients are evaluated with regard to recurrence following surgery. Eight patients had relapsed and thirteen patients did not for a period of 4 years after the surgical procedure. The same processing steps as PROS is used.

##### Lymphoma

Diffuse large B-cell lymphoma (DLBCL) is the most common lymphoid malignancy in adults, curable in less than 50% of patients. This data-set is constructed from a related germinal center B-cell, follicular lymphoma (FL) [5]. In DLBCL-FL, the microarray contains gene expression profiles for 77 patients (58 with DLBCL and 19 with FL) for a total of 6817 genes. Accepting the suggestion of [5], we use the value of 16, 000 as a ceiling and 20 as the lower threshold for the expression levels. The variation filter is used to exclude genes showing small variation across samples. Two types of variations are used here: fold-change and absolute variation, which correspond to *max/min *and *max *- *min*, respectively; where *max *and *min *refer to the maximum and minimum value of expression level for each particular gene across all samples. In particular, we used *max/min *< 3 and *max *- *min *< 100.

##### Leukemia

[1] define a data-set for the study of two types of acute leukemia – acute lymphoblastic leukemia (ALL) and acute myeloid leukemia (AML). The microarrays contain 6817 genes. The data used in this paper consists of 38 bone marrow samples (27 ALL and 11 AML). The leave-one-out cross-validation test was used on this set. The same filtering procedure defined above was employed.

##### I2000

This data-set contains a total of 2000 gene expressions of 40 tumor and 22 normal colon tissue samples [3]. Following the suggestion of [4], we employed the following preprocessing: 1) compute the median of each array (an array corresponds to a specimen); 2) determine the median of the medians computed in step 1, which is labelled *M*; 3) for a given array, add or subtract an appropriate constant to each expression value to re-center the median of the array to be that given by *M*; 4) log-transform the entire data-set to make the data more Gaussian distributed.

#### Experimental results

To test the approach just presented, we use the leave-one-out cross-validation test. This means that, at each iteration, we keep one of the *n *samples for testing and use the remaining *n *- 1 for training. We then see whether each of the algorithms can correctly classify the sample left out. This is repeated *n *times – one for each of the samples that can be left out. Table 1 shows the results obtained using the proposed approach on a diverse set of microarray cancer classification problems. In the table, we also show comparisons to the classical approaches mentioned above: *k*NN, WV, LDA, SVM, and ML.

**Table 1:** Comparison of the results obtained with different classifiers in a variety of data-sets.

Data-set	genes	samples	DP	*k*NN	WV	LDA	SVM	ML-s	ML-d
BRCA1	3226	7 BRCA1-positive	**21/22**	18/22 (1)	18/22	18/22	18/22	19/22	16/22
		15 BRCA1-negative							
BRCA2	3226	8 BRCA2-positive	**21/22**	**21/22 **(1)	17/22	19/22	18/22	17/22	17/22
		14 BRCA2-negative							
PROS	12600	52 tumor tissue	**93/102**	90/102 (5)	61/102	92/102	**93/102**	64/102	50/102
		50 normal tissue							
PROS-OUT	12625	8 non-recurrence	**15/21**	12/21 (1)	12/21	13/21	14/21	13/21	13/21
		13 recurrence							
DLBCL-FL	6817	52 DLBCL	**74/77**	71/77 (7)	63/77	**74/77**	**74/77**	65/77	58/77
		25 FL							
ALL-AML	6817	27 AML	**38/38**	37/38 (3)	**38/38**	**38/38**	**38/38**	30/38	27/38
		11 ALL							
I-2000	2000	40 tumor colon tissue	**61/62**	59/62 (3)	58/62	**61/62**	**61/62**	59/62	58/62
		22 normal colon tissue							

Columns indicate the algorithm used, rows the data-set. In each cell the number in the numerator specifies the number of left-out-samples that has been correctly classified by the corresponding algorithm. The value in the denominator is the total number of samples *n*. The *k*NN algorithm has a free parameter that needs to be determined – the number of neighbors *k*. To allow for a fair comparison, we have optimized this value for each of the databases using cross-validation [12]. The optimal resulting value is specified in parenthesis. In the ML classifier, we consider two cases: those where the two classes are assumed to have the same variance, and those where the variances are assumed to be different. These are referred to as ML-s (same) and ML-d (different).

The second experiment is designed to further understand the dynamics of the proposed algorithm. As stated in this paper, most algorithms will fail when the training samples are not representative of the testing ones. We have approached this problem by taking advantage of the information embedded in the test vector. Under this model, our approach should be superior to the classification mean of other algorithms when the test sample is more correlated to the training samples of the incorrect class. To demonstrate this, we have designed a second experiment, where we divided each of the samples left out for testing into two groups. The first group includes those test vectors that are more correlated to the sample mean of the correct class than to the sample mean of the incorrect class. The second set corresponds to those test samples that are more correlated to the incorrect class. Table 2 shows the classification accuracy of our method on each of these two sets for each of the databases tested. Our results are compared to the average of those obtained with the other algorithms tested. As predicted, the largest differences are in the second group, which includes the test vectors that are more correlated to the incorrect class.

**Table 2:** Classification accuracy of the proposed algorithm and alternatives on two subsets of the data in the leave-one-out test.

	BRCA1		BRCA2		PROS		PROS-OUT	
	
	DP	Others	DP	Others	DP	Others	DP	Others
More correlated	18/18	16.67/18	17/17	16.5/17	59/60	53.3/60	12/15	11.83/15
Less correlated	3/4	1.67/4	4/5	1.67/5	34/41	21.67/41	3/6	0.9/6

	DLBCL-FL		ALL-AML		I2000			
			
	DP	Others	DP	Others	DP	Others		

More correlated	62/62	58.33/62	38/38	34.67/38	58/58	57.83/58		
Less correlated	12/15	9.17/15	0/0	0/0	3/4	1.5/4		

The first subset includes those test feature vectors that are more correlated to the samples of the correct class (called, more correlated in this table). The second subset consists of those test feature vectors that are more correlated to the samples of the incorrect class (referred to as less correlated). The proposed approach is superior in both subsets, but especially so in the less correlated category. This is achieved by taking advantage of the information encoded in the test sample.

The experimental results reported thus far used real datasets to compare the classification capabilities of the proposed algorithm with those reported in the literature. The differences shown in Tables 1 and 2 are significant, because our method is able to provide the top classification accuracies in all cases. Yet, one may wonder how would our method perform if the availability of samples was larger. To further demonstrate the superiority of the proposed algorithm with those given in the literature, we now show an experimental comparison using synthetic datasets.

**Figure 4:**
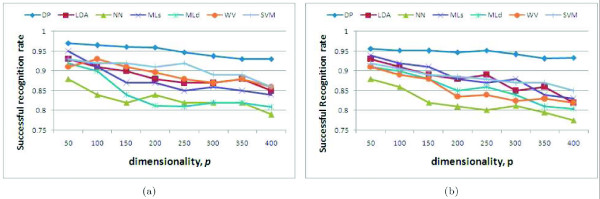
**Experimental results with synthetic data.** Shown here are the successful recognition rates obtained with a variety of classification approaches defined in the literature for increasing values of the dimensionality *p *– ranging from a low of 50 to a maximum of 400. Recognition rates shown in the scale of 0 to 1. (a) Results generated with a single Gaussian distribution per class. (b) Results obtained with samples randomly drawn from a mixture of four randomly generated Gaussian distribution per class.

In our first example, we randomly generated two Gaussian distribution, each representing one of the two classes. The two Gaussians were defined in ℝ^*p*^, with *p *= 50,...,400. The covariance matrices were set as diagonal matrices with their elements set at random. The means of these distributions were also set at random. We then randomly generated 50 samples from each of the two distribution and used the algorithms defined earlier and the one proposed in the present work to do classification. The number of samples was kept at 50 regardless of value of *p*. This tested how well each algorithm could deal with a decrease on the sample-to-dimension ratio. In Fig. 4(a), we plot the average results obtained from a total of 100 randomly generated cases. We clearly see that the proposed *DP *algorithm outperform the others – especially so as *p *increases.

The above result is however quite simplistic, because the samples in each distribution were distributed according to a single Gaussian distribution. A more realistic scenario in bioinformatics is that where the samples in each class are generated by a mixture of Gaussians. To test this other case, we randomly generated 50 samples corresponding to two different classes. Each class was now defined by a mixture of four Gaussians, with their means and covariances randomly selected as above. The average results over a total of 100 runs are shon in Fig. 4(b). Again, we see that the proposed *DP *algorithm outperforms the others. *Most importantly *though, it is clear from Fig. 4(a–b) that the proposed algorithm is not very sensitive to an increase on the number of dimensions. This is a very important feature in studies of bioinformatics and further demonstrates the superiority of the *DP *algorithm over the state of the art.

## Discussion

Analyzing data from small sample size sets is a recurring problem in biology. This is generally due to the limited amount of data available or to the difficulty or costs associated to obtaining additional data. Studies indicate that hundreds or thousands of samples would be required to extract useful statistical information from our data sets [15,16]. Hence, innovative statistical methods like the one presented in this paper are of great relevance in many areas of biology.

This paper has shown derivations of an approach to deal with the small sample size problem within a linear discriminant analysis setting. Our framework can be readily extended to work within other classification approaches. It could also be combined with shrinkage [28], a mechanism to share information between genes, to improve on the analysis of our data. A key point is to realize that (in our framework) it is not necessary to learn the true, underlying distribution of each class. It suffices to find that (part of the) solution necessary to correctly classify the test sample. Part of this information is of course embedded in the test sample, and our approach takes advantage of this. While our results are most applicable to data-sets where the data in each class can be approximated by an underlying distribution, data-drive approaches may be preferred elsewhere. Our framework should then be extended into other algorithms such as non-parametric methods or SVM [23]. Extensions to deal with missing components [29,30] can also be adapted to our framework. Also, some genome sequences are spherical. In these cases, our approach can be extended to work with spherical classifiers [31].

The approach proposed here can also be applied to many other problems in biology and medicine. For example, in the classification of nuclear magnetic resonance spectra, which is typically used to carry out metabolomics experiments. In this example, classification approaches like the ones describe din this paper are generally used [32]. Another application is in the use of cytotoxic chemotherapeutic drugs that target proliferating signature genes. This approach is generally used to stop further cell division and bring tumors under control. However, these drugs can also damage DNA of normal tissue. Developing solutions that only target those necessary genes is fundamental to the success of such therapies. This will involve the identification of biomarkers of proliferation associated to each of the cancers [33]. These analysis are also characterized by a disproportionate feature to sample ratio, resulting in over-fitting. This is especially true when proliferation is studied over a large number of cancers [34,35]. In such studies it is almost always necessary to use all the data available to prevent missing useful biomarkers.

## Authors' contributions

The two authors contributed equally in the development of the approach and the writing of the paper. MZ generated the results.
